# The 32nd summer school of the Research Community for Mechanisms of Mutations

**DOI:** 10.1186/s41021-019-0134-7

**Published:** 2019-11-12

**Authors:** Masanobu Kawanishi

**Affiliations:** 1 Secretary general, the Research Community for Mechanisms of Mutations, Osaka, Japan; 2 Graduate School of Science and Radiation Research Center, Osaka Prefecture University, 1-2 Gakuen-cho, Nakaku, Sakai-city, Osaka, 599-8570 Japan

**Keywords:** Mutagenesis, Carcinogenesis, Genotoxicity, The research Community for Mechanisms of mutations, The Japanese environmental mutagen society, Young scientists

## Abstract

The 32nd summer school of the Research Community for Mechanisms of Mutations was held at Inter-University Seminar House in Hachioji city, Tokyo, from September 7 to 8, 2019. Thirty-eight people attended this annual event, and three eminent researchers were invited to discuss DNA damage induced by endogenous aldehydes, “action-at-a-distance mutagenesis” and a novel genome editing method, and DNA repair in fungi and plants. In addition to these plenary sessions, eleven participants presented their own research in oral sessions. More than half of the participants were young scientists such as graduate/undergraduate students, post-doctoral fellows and assistant professors. All members joined in enthusiastic discussions and acquired new scientific knowledge through these two days.

The 32nd summer school of the Research Community for Mechanisms of Mutations (RCMM) was held at the Inter-University Seminar House in Hachioji city, Tokyo, from September 7 to 8, 2019. The host of the 32nd summer school was Prof. Tatsuo Nunoshiba (International Christian University). The RCMM has organized a conference annually since it was launched as the Society for Mechanisms of Anti-mutagenesis and Anti-carcinogenesis Studies (SMAAS) by members of the Japanese Environmental Mutagen Society (JEMS) in 1987 [1]. The SMAAS developed into the RCMM in 2006, and since then the Community has held a conference entitled “summer school” each year [1]. The aim of the summer school is to provide an occasion to learn about mutation research frontiers and to exchange scientific information, especially among young scientists such as graduate/undergraduate students, post-doctoral fellows and assistant professors. In 2019, thirty-eight people attended the 32nd summer school, and three eminent researchers were invited to discuss their current research at the plenary sessions:

Dr. Jun Nakamura (Osaka Prefecture University) presented “Endogenous aldehydes, from DNA metabolism to metabolic syndrome”. Having developed a highly sensitive apurinic/apyrimidinic (AP) site quantification method and “real-time” single strand break (SSB) detection assay [2, 3], he estimated the frequencies of spontaneous or induced AP site and SSB in mammalian cells using these methods. He also introduced cluster DNA damage induced by H_2_O_2_, and the finding that Fanconi anemia group D2 protein (FANCD2) repairs DNA crosslinks induced by endogenous aldehyde. In addition to these scientific discoveries, introducing his career in the United States of America after leaving a Japanese pharmaceutical company, he encouraged young Japanese students to leap into the world with an ever-challenging spirit.

Dr. Hiroyuki Kamiya (Hiroshima University) presented “Action-at-a-distance mutagenesis and development of a novel genome editing method without artificial endonucleases”. He introduced a shuttle vector plasmid site-specifically modified with single 8-oxo-guanine into human cells in which Werner syndrome protein (WRN) was knocked down, and found replicated plasmids with base-substitution mutations at various positions, suggesting that the reduction of the WRN induced action-at-a-distance mutagenesis [4]. In contrast, when the plasmid site-specifically modified with an AP site was replicated, the action-at-a-distance mutagenesis was observed in even WRN-proficient cells. He also presented his novel genome editing method without artificial endonucleases to avoid the induction of DNA strand breaks at off-target sites [5, 6].

Dr. Ryouhei Yoshihara (Saitama University) presented “Analysis of biological phenomena associated with DNA repair in fungi and plants”. He identified a gene, of which a defect causes a short-lifespan, by screening a *Neurospora crassa* knockout library [7]. He discovered the protein encoded by the gene, named mitochondrial high mobility group box protein 1 (MHG1), plays an important role in the maintenance of mitochondrial DNA, and speculated that mitochondrial abnormality caused by mitochondrial DNA aberration is responsible for the short-lifespan of the strain [7]. He also introduced his study on involvement of non-homologous end joining (NHEJ) of plant cells with the gene transfer process of Agrobacterium infection.

In addition to these plenary sessions, eleven investigators presented their own research in oral sessions. Participants in the summer school spanned several generations, from undergraduate students in their twenties to professors emeritus in their seventies. More than half were students and young scientists seeking their research interest and/or the meaning of their lives. All members took part in enthusiastic discussions and acquired new knowledge through these two days. The author believes that these activities contributed to the achievement of meaningful lives as well as the progress of scientific research.

The 33rd summer school will be held at the same venue, Inter-University Seminar House in Hachioji city, Tokyo, from September 12 to 13, 2020.

## Conclusion

The 32nd summer school organized by RCMM in September 2019 finished successfully. Thirty-eight people participated in the event and actively discussed many topics. Three invited scientists presented their recent research in DNA repair and mutagenesis, and eleven researchers gave talks on their own scientific achievements. All members enjoyed hearing and discussing recent advances in mutagenesis and related research fields, and communicating between and among their institutions. The students and young scientists were stimulated and encouraged to make great progress in the near future. The 33rd summer school will be held at the same venue, Inter-University Seminar House in Hachioji city, Tokyo, from September 12 to 13, 2020.

## Data Availability

Not applicable.

## Abbreviations

AP: Apurinic/apyrimidinic
FANCD2: Fanconi anemia group D2 protein
JEMS: Japanese Environmental Mutagen Society
MHG1: Mitochondrial high mobility group box protein 1
NHEJ: Non-homologous end joining
RCMM: The Research Community for Mechanisms of Mutations
SMAAS: The Society for Mechanisms of Anti-mutagenesis and Anti-carcinogenesis Studies
SSB: Single strand break
WRN: Werner syndrome protein
